# Supplementary material for: Smooth Interpolating Curves with Local Control and Monotone Alternating Curvature
Source: Comput Graph Forum. 2022 Oct 6;41(5):25–38. doi: 10.1111/cgf.14600 (PMC9827861; doi:10.1111/cgf.14600)
Supplement: Supplementary file 1 — Supplement Material [file CGF-41-25-s001.zip › Local-Smooth-Interpolating-MonoCurvature/extern/clothoids/submodules/Utils/docs/api-cpp/program_listing_file_Utils_Table.hxx.html]

Program Listing for File Table.hxx — Utils v1.2

### Navigation

- index
- toc
- Utils »
- Program Listing for File Table.hxx

# Program Listing for File Table.hxx¶

↰ Return to documentation for file (`Utils/Table.hxx`)

```
/*--------------------------------------------------------------------------*\
 |                                                                          |
 |  Copyright (C) 2021                                                      |
 |                                                                          |
 |         , __                 , __                                        |
 |        /|/  \               /|/  \                                       |
 |         | __/ _   ,_         | __/ _   ,_                                |
 |         |   \|/  /  |  |   | |   \|/  /  |  |   |                        |
 |         |(__/|__/   |_/ \_/|/|(__/|__/   |_/ \_/|/                       |
 |                           /|                   /|                        |
 |                           \|                   \|                        |
 |                                                                          |
 |      Enrico Bertolazzi                                                   |
 |      Dipartimento di Ingegneria Industriale                              |
 |      Universita` degli Studi di Trento                                   |
 |      email: enrico.bertolazzi@unitn.it                                   |
 |                                                                          |
\*--------------------------------------------------------------------------*/


/*\

  Based on terminal-table:

  https://github.com/Bornageek/terminal-table

  Copyright 2015 Andreas Wilhelm

  Licensed under the Apache License, Version 2.0 (the "License");
  you may not use this file except in compliance with the License.
  You may obtain a copy of the License at

      http://www.apache.org/licenses/LICENSE-2.0

  Unless required by applicable law or agreed to in writing, software
  distributed under the License is distributed on an "AS IS" BASIS,
  WITHOUT WARRANTIES OR CONDITIONS OF ANY KIND, either express or implied.
  See the License for the specific language governing permissions and
  limitations under the License.

\*/

#pragma once

#ifndef DOXYGEN_SHOULD_SKIP_THIS
#ifndef UTILS_TABLE_dot_HXX
#define UTILS_TABLE_dot_HXX
#endif

namespace Utils {

  namespace Table {

    using std::string;
    using std::vector;

    class Table;

    typedef int integer;

    enum Alignment { LEFT, RIGHT, CENTER };

    class Style {
    private:

      char m_BorderTop         = '-';
      char m_BorderTopMid      = '+';
      char m_BorderTopLeft     = '+';
      char m_BorderTopRight    = '+';

      char m_BorderBottom      = '-';
      char m_BorderBottomMid   = '+';
      char m_BorderBottomLeft  = '+';
      char m_BorderBottomRight = '+';

      char m_BorderLeft        = '|';
      char m_BorderLeftMid     = '+';

      char m_BorderMid         = '-';
      char m_BorderMidMid      = '+';

      char m_BorderRight       = '|';
      char m_BorderRightMid    = '+';

      char m_BorderMiddle      = '|';

      integer m_PaddingLeft    = 1;
      integer m_PaddingRight   = 1;

      Alignment m_Align = Alignment::LEFT;

      integer m_Width = 0;

    public:

      Style() {}

      char borderTop() const { return m_BorderTop; }
      void borderTop( char borderStyle ) { m_BorderTop = borderStyle; }

      char borderTopMid() const { return m_BorderTopMid; }
      void borderTopMid( char borderStyle ) { m_BorderTopMid = borderStyle; }

      char borderTopLeft() const { return m_BorderTopLeft; }
      void borderTopLeft( char borderStyle ) { m_BorderTopLeft = borderStyle; }

      char borderTopRight() const { return m_BorderTopRight; }
      void borderTopRight( char borderStyle ) { m_BorderTopRight = borderStyle; }

      char borderBottom() const { return m_BorderBottom; }
      void borderBottom( char borderStyle ) { m_BorderBottom = borderStyle; }

      char borderBottomMid() const { return m_BorderBottomMid; }
      void borderBottomMid( char borderStyle ) { m_BorderBottomMid = borderStyle; }

      char borderBottomLeft() const { return m_BorderBottomLeft; }
      void borderBottomLeft( char borderStyle ) { m_BorderBottomLeft = borderStyle; }

      char borderBottomRight() const { return m_BorderBottomRight; }
      void borderBottomRight( char borderStyle) { m_BorderBottomRight = borderStyle; }

      char borderLeft() const { return m_BorderLeft; }
      void borderLeft( char borderStyle ) { m_BorderLeft = borderStyle; }

      char borderLeftMid() const { return m_BorderLeftMid; }
      void borderLeftMid( char borderStyle ) { m_BorderLeftMid = borderStyle; }

      char borderMid() const { return m_BorderMid; }
      void borderMid( char borderStyle ) { m_BorderMid = borderStyle; }

      char borderMidMid() const { return m_BorderMidMid; }
      void borderMidMid( char borderStyle ) { m_BorderMidMid = borderStyle; }

      char borderRight() const { return m_BorderRight; }
      void borderRight( char borderStyle ) { m_BorderRight = borderStyle; }

      char borderRightMid() const { return m_BorderRightMid; }
      void borderRightMid( char borderStyle ) { m_BorderRightMid = borderStyle; }

      char borderMiddle() const { return m_BorderMiddle; }
      void borderMiddle( char borderStyle ) { m_BorderMiddle = borderStyle; }

      integer paddingLeft() const { return m_PaddingLeft; }
      void paddingLeft( integer padding ) { m_PaddingLeft = padding; }

      integer paddingRight() const { return m_PaddingRight; }
      void paddingRight( integer padding ) { m_PaddingRight = padding; }

      Alignment alignment() const { return m_Align; }
      void alignment( Alignment align ) { m_Align = align; }

      integer width() const { return m_Width; }
      void width( integer width ) { m_Width = width; }
    };

    class Cell {
    private:
      Table *   m_Table   = nullptr;
      string    m_Value   = "";
      Alignment m_Align   = Alignment::LEFT;
      integer   m_ColSpan = 1;
      integer   m_Width   = 0;

    public:

      Cell() {}

      Cell(
        Table*         table,
        string const & val = "",
        integer        colSpan = 1
      );

      string const & value() const { return m_Value; }
      void value( string const & val ) { m_Value = val; }

      Alignment alignment() const { return m_Align; }
      void alignment( Alignment align ) { m_Align = align; }

      integer colSpan() const { return m_ColSpan; }
      void colSpan( integer colSpan ) { m_ColSpan = colSpan; }

      integer width( integer col ) const;
      integer height() const;

      integer maxLineWidth() const;

      string line( integer idx ) const;
      void trimLine( string & line ) const;

      string render( integer line, integer col ) const;
    };

    class Row {
    protected:
      typedef vector<Cell>             vecCell;
      typedef std::vector<std::string> vecstr;

      Table * m_Table = nullptr;
      vecCell m_Cells;

    public:

      Row() {}

      Row(
        Table *        table,
        vecstr const & cells = vecstr()
      );

      Table const * table() const { return m_Table; }

      //vecCell & cells() { return m_Cells; }
      void cells( vecstr const & cells );

      integer numCells() const { return integer(m_Cells.size()); }
      integer cellWidth( integer idx ) const;
      void cellColSpan( integer idx, integer span );

      void cell( string const & value );
      //Cell& cell( integer idx ) { return m_Cells[idx]; }

      Cell const & operator [] ( integer idx ) const { return m_Cells[idx]; }
      Cell       & operator [] ( integer idx )       { return m_Cells[idx]; }

      integer height() const;

      string render() const;
    };

    class Table {
    public:
      typedef vector<Row>    vecRow;
      typedef vector<Cell>   vecCell;
      typedef vector<string> vecstr;
      typedef vector<vecstr> vecvecstr;
      typedef int integer;

    private:
      Style  m_Style;
      string m_Title;
      Row    m_Headings;
      vecRow m_Rows;

    public:

      Table() {}

      Table(
        Style     const & style,
        vecvecstr const & rows = vecvecstr()
      )
      : m_Style(style) {
        this->rows(rows);
      }

      void
      setup(
        Style     const & style,
        vecvecstr const & rows = vecvecstr()
      ) {
        m_Style = style;
        this->rows(rows);
      }

      void alignColumn( integer n, Alignment align );
      void addRow( vecstr const & row );

      integer cellSpacing() const;
      integer cellPadding() const;

      vecCell column( integer n ) const;
      integer columnWidth( integer n ) const;
      integer numColumns() const;

      Style const & style() const { return m_Style; }

      void style( Style const & style ) { m_Style = style; }

      string const & title() const { return m_Title; }

      void title( string const & title ) { m_Title = title; }

      Row const & headings() const { return m_Headings; }

      void headings( vecstr const & headings );

      Row       & row( integer n );
      Row const & row( integer n ) const;

      Row       & operator [] ( integer n )       { return this->row(n); }
      Row const & operator [] ( integer n ) const { return this->row(n); }

      Cell       & operator () ( integer i, integer j )       { return (*this)[i][j]; }
      Cell const & operator () ( integer i, integer j ) const { return (*this)[i][j]; }

      vecRow const & rows() const { return m_Rows; }
      void rows( vecvecstr const & rows );

      string
      renderSeparator(
        char const left,
        char const mid,
        char const right,
        char const sep
      ) const;

      string render() const;
    };
  }
}

inline
Utils::ostream_type&
operator << ( Utils::ostream_type& stream, Utils::Table::Row const & row ) {
  return stream << row.render();
}

inline
Utils::ostream_type&
operator << ( Utils::ostream_type& stream, Utils::Table::Table const & table ) {
  return stream << table.render();
}

#endif
```

### Quick search

### Table of Contents

- rang - Colors for your Terminal
- Zstr
- C++ API

«
hide menu

menu
sidebar
»

### Navigation

- index
- toc
- Utils »
- Program Listing for File Table.hxx

© Copyright 2021, Enrico Bertolazzi.
Created using Sphinx 4.2.0.
